# Health Tract, No. 210

**Published:** 1865

**Authors:** 


HEALTH TRACT, No. 210.

### CHEAP BREAD.

"BREAD and butter" are the only articles of food of which we never tire for a day, from early childhood to extreme old age. A pound of fine flour or Indian (corn meal) contains three times as much nutriment as one pound of butcher's roast beef; and if the whole product of the grain, bran, and all, were made into bread, fifteen per cent more of nutriment would be added. Unfortunately, the bran, the coarsest part, is thrown away; the very part which gives soundness to the teeth and strength to the bones, and vigor to the brain. Five hundred pounds of fine flour give to the body thirty pounds of the bony element; while the same quantity of bran gives one hundred and twenty-five pounds! This bone is "lime," the phosphate of lime; the indispensable element of health to the whole human body; from the want of the natural supply of which multitudes of persons go into a general "decline." But swallowing "phosphates" in the shape of powders, or in syrups, to cure these "declines," has little or no virtue. The articles containing these "phosphates" must pass through nature's laboratory; must be subject to her manipulations, in alembics specially prepared by Almighty power and skill, in order to impart their peculiar virtues to the human frame; in plainer phrase, the shortest, safest, and most infallible method of giving strength to body, bone, and brain, thereby arresting disease, and building up the constitution, is to eat and digest more bread made out of the whole grain, whether of wheat, corn, rye, or oats. But we must get an appetite for eating more, and a power of digesting more. Not by the artificial and lazy method of drinking bitters and taking tonics, but by moderate, continued, and remunerative muscular exercise in the open air every day, rain or shine. And that we may eat the more of it, the bread must be good, and cheap, and healthful; and that which combines these three qualities to a greater extent than any other known on the face of the globe, as far as we know, is made thus: To two quarts of corn (Indian) meal, add one pint of bread-sponge, water sufficiently to wet the whole; add one half pint of flour and a teaspoonful of salt. Let it rise; then knead well, unsparingly, for the second time. Place the dough in the oven, and let it bake an hour and a half. Keep on trying until you succeed in making a light, well-baked loaf. Our cook succeeded admirably by our directions at the very first trial. It costs just half as much as bread from the finest family flour, is lighter on the stomach, and imparts more health, vigor, and strength to body, brain, and bone. Three pounds of such bread, (at five cents a pound for the meal,) certainly affords as much nutriment as nine pounds of good roast beef, (costing at twenty-five cents $2.25,) according to standard physiological tables.

